# Effect of Dietary Zinc Methionine Supplementation on Growth Performance, Immune Function and Intestinal Health of Cherry Valley Ducks Challenged With Avian Pathogenic *Escherichia coli*

**DOI:** 10.3389/fmicb.2022.849067

**Published:** 2022-05-03

**Authors:** Yaqi Chang, Jia Mei, Ting Yang, Zhenyu Zhang, Guangmang Liu, Hua Zhao, Xiaoling Chen, Gang Tian, Jingyi Cai, Bing Wu, Fali Wu, Gang Jia

**Affiliations:** ^1^Key Laboratory for Animal Disease-Resistance Nutrition of China, Ministry of Education, Institute of Animal Nutrition, Sichuan Agricultural University, Chengdu, China; ^2^Institute of Animal Husbandry and Veterinary Science, Jiangxi Academy of Agricultural Sciences, Nanchang, China; ^3^Institute of Animal Husbandry and Veterinary Medicine, Meishan Vocational Technical College, Meishan, China; ^4^Chelota Group, Guanghan, China

**Keywords:** zinc methionine, avian pathogenic *Escherichia coli* (APEC), growth performance, intestinal immune, meat ducks

## Abstract

This study was carried out to evaluate the effects of supplemental zinc methionine (Zn–Met) on growth performance, immune function, and intestinal health of meat ducks challenged with avian pathogenic *Escherichia coli* (APEC). A total of 480 1-day-old Cherry Valley male ducks were randomly assigned to 8 treatments with 10 replicates, each replicate containing 10 ducks. A 4 × 2 factor design was used with four dietary zinc levels (0, 30, 60, 120 mg Zn/kg in the form Zn–Met was added to the corn–soybean basal diet) and challenged with or without APEC at 8-days-old ducks. The trial lasted for 14 days. The results showed that a dietary Zn–Met supplementation significantly increased body weight (BW) of 14 days and BW gain, and decreased mortality during 7–14-days-old ducks (*p* < 0.05). Furthermore, dietary 30, 60, 120 mg/kg Zn–Met supplementation noticeably increased the thymus index at 2 days post-infection (2 DPI) and 8 DPI (*p* < 0.05), and 120 mg/kg Zn–Met enhanced the serum IgA at 2 DPI and IgA, IgG, IgM, C3 at 8 DPI (*p* < 0.05). In addition, dietary 120 mg/kg Zn–Met supplementation dramatically increased villus height and villus height/crypt depth (*V*/*C*) of jejunum at 2 DPI and 8 DPI (*p* < 0.05). The *TNF-*α and *IFN-*γ mRNA expression were downregulated after supplemented with 120 mg/kg Zn–Met in jejunum at 8 DPI (*p* < 0.05). Moreover, dietary 120 mg/kg Zn–Met supplementation stimulated *ZO-3*, *OCLN* mRNA expression at 2 DPI and *ZO-2* mRNA expression in jejunum at 8 DPI (*p* < 0.05), and improved the MUC2 concentration in jejunum at 2 DPI and 8 DPI (*p* < 0.05). At the same time, the cecal *Bifidobacterium* and *Lactobacillus* counts were increased (*p* < 0.05), and *Escherichia coli* counts were decreased (*p* < 0.05) after supplemented with Zn–Met. In conclusion, inclusion of 120 mg/kg Zn–Met minimizes the adverse effects of APEC challenge on meat ducks by improving growth performance and enhancing immune function and intestinal health.

## Introduction

Colibacillosis caused by *Escherichia coli* is one of the most common and important diseases in poultry industry as it leads to reduced weight gain, increased mortality, and reduced flock uniformity (Nolan et al., 2020), causing serious economic losses more than 5.7 billion pounds for producers in 2017 (National Agricutural Statistics Service [NASS] And USDA, 2017; Wibisono et al., 2018). Although *E. coli* as a normal inhabitant can be found in the intestinal tract of birds, certain strains can cause respiratory and systemic diseases that known as avian pathogenic *Escherichia coli* (APEC) (Guabiraba and Schouler, 2015). Respiratory tract and oral cavity are the initial sites where APEC enters the whole body. The body recognizes the pathogen-associated molecular patterns (PAMPs) on APEC through Toll-like receptors (TLRs), thus inducing immune response (Roy and Mocarski, 2007). Antibiotics usually used to prevent and control colibacillosis, but the long-term use of antibiotics leads to antibiotic residues in poultry products and the emergence of multidrug-resistant bacteria, making the prevention of colibacillosis more difficult (Yuvraj, 2019). Therefore, how to prevent and control the negative impact of *E. coli* on the meat duck industry has become a problem that needs to be studied.

Zinc is an essential micronutrient with multiple effects on enzymatic reactions, DNA synthesis and many cellular functions. Its biological functions include promoting animal growth and development, enhancing antioxidant and immune capacity, and improving the quality of poultry products and intestinal health (Chien et al., 2006; Liu et al., 2011). Emerging research shows that zinc has an important function in protecting from infection of pathogens. Zhang et al. (2012) showed that zinc alleviates intestinal injury by increasing the ileal villi height and V/C ratio while upregulating the expression of *Occludin* and *Claudin-1* mRNA in *Salmonella*-infected broilers. Besides, zinc supplementation increased the populations of *Lactobacillus* and decreased epithelium cell apoptosis index in broilers challenged with *Salmonella* (Shao et al., 2014). Inorganic zinc, such as ZnO and ZnSO_4_, is mainly supplemented in the diet to meet the zinc requirements of animals due to its low price. In comparison, organic zinc exhibited more stable biochemical properties, higher bioavailability and better resistance to infection (Esfahani et al., 2021). Excessive supply of Zn sulfate (ZnSO_4_, 120–240 mg/kg diet) more than the Zn requirement (60 mg/kg diet) recommended by the National Research Council (National Research Council, 1994) in diets designed for ducks is used to promote growth and intestinal health, possibly by modulating the integrity of intestinal barrier function (Wen et al., 2018). Zinc methionine (Zn–Met), as an organic zinc source, is better than inorganic zinc in improving immunity and anti-stress (Kidd et al., 1994; Saleh et al., 2018). Saleh et al. (2018) found that dietary Zn–Met enhances humoral immune response and antioxidant enzyme activity of heat stressed broilers. Wei (2019) reported that supplemented with Zn–Met posed to anti-diarrhea and growth promotion by enhancing average daily gain (ADG) and reducing diarrhea rate and serum D-lactate content, meanwhile upregulating jejunal tight junction protein mRNA expression of *Occludin* and *Claudin-1*, *ZO-1* mRNA in newborn calves challenged with *E. coli* K88. In addition, zinc can reduce the expression of *E. coli* virulence factors, inhibit bacterial attachment and internalization, destroy cell membrane and lead to cell content leakage, and finally kill *E. coli* (Liu et al., 2009). Zinc can effectively eliminate *E. coli* by inducing the expression of antibacterial substances such as antibacterial peptide (Talukder et al., 2011). However, up to now, there has been no study examining the use of dietary Zn–Met supplementation and its effects on infected meat ducks. Therefore, the objective of the current experiment was to investigate the effects of supplementation of Zn–Met on growth performance, immune function, and intestinal health in meat ducks challenged with APEC.

## Materials and Methods

### Birds, Experimental Design, Diets, and Management

Animals, diets and experimental design, care handling, and sampling procedures were approved by the Animal Welfare Committee of Sichuan Agricultural University (No. 20180718) before initiation of the trial. A total of 480 1-day-old healthy Cherry Valley male ducks were obtained from a commercial hatchery and randomly divided into 8 treatments according to the principle of no difference in body weight (BW), each with 6 replicate pens of 10 ducks. The study adopted a completely randomized design with a 4 × 2 factorial pattern. Herein, four levels of Zn–Met supplementation (0, 30, 60, and 120 mg/kg) and APEC challenge (with or without), experimental treatments (diets) include (1) control diet (U0); (2) control + 30-mg/kg Zn–Met (U30); (3) control + 60-mg/kg Zn—Met (U60); (4) control + 120-mg/kg Zn–Met (U120); (5) control + APEC (C0); (6) control + APEC + 30-mg/kg Zn–Met (C30); (7) control + APEC + 60-mg/kg Zn–Met (C60); (8) control + APEC + 120-mg/kg Zn–Met (C120). The addition of graded Zn–Met caused the different content of methionine and we corrected it by addition of extra methionine in the premixes. Hence, all nutrients were kept at the same levels expect for the zinc content.

The ducks were raised for 14 days in cages (1.00 m × 1.00 m × 0.55 m) in a temperature- and humidity-controlled room with a continuous 24-h light supply. All ducks had *ad libitum* access to feed and water throughout the experiment period. The temperature and humidity control process according to the feeding standard of meat ducks (NY/T 2122–2012). A corn–soybean basal diet based on National Research Council (1994) was formulated to meet the requirement expect zinc of meat ducks (Table 1). Dietary treatments included the basal diet supplemented with zinc methionine (Chelota, Guanghan, China) at levels of 0, 30, 60, 120 mg/kg (as Zn). The measured values of zinc contained in diets were 27.89, 58.47, 89.31, and 151.55 mg/kg. Different levels of methionine were added exogenously to ensure consistent methionine levels in the diets of each treatment group.

**TABLE 1:** Composition and nutrient levels of the basic diets for meat ducks (air-dry basis).

Ingredient	Ratio (%)	Nutrient levels	Calculated values
Corn	59.20	AME (MJ/kg)	12.15
Soybean oil	0.50	Crude protein (%)	20.14
Soybean meal	34.75	Ca (%)	0.98
Wheat bran	1.50	Available P (%)	0.42
CaCO_3_*^a^*	0.98	Digestible lysine (%)	1.11
CaHPO_4_⋅2H_2_O*^a^*	2.04	Digestible methionine (%)	0.46
NaCl*^a^*	0.30	Digestible Met + Cys (%)	0.79
Choline chloride (50%)*^a^*	0.15	Digestible threonine (%)	0.85
Vitamin premix*^b^*	0.03	Digestible tryptophan (%)	0.32
Mineral premix*^c^*	0.20	Analyzed level	
*L*-Lysine.HC1 (98%)*^a^*	0.03	Zinc/(mg/kg)	27.89
*DL*-Methionine*^a^*	0.15		
*L*-tryptophan*^a^*	0.09		
*L*-threonine*^a^*	0.08		
Total	100		

*^a^Feed grade.*

*^b^Contained the following per kilogram of the diet: VA 9000 IU, VD_3_ 2000 IU, VE 10 mg, VB_1_ 2 mg, VB_2_ 4.8 mg, VB_3_ 50 mg, VB_5_ 20 mg, VB_9_ 1 mg, VB_12_ 0.02 mg.*

*^c^Cu (CuSO_4_⋅5H_2_O) 8 mg, Fe (FeSO_4_⋅H_2_O) 80 mg, Mn (MnSO_4_⋅H_2_O) 70 mg, Se (NazSeO3) 0.3 mg, I (KI) 0.4 mg.*

### Avian Pathogenic *Escherichia coli* Challenge

The strain used in this experiment is APEC (serotype: O78) of duck-origin supplied from College of Veterinary Medicine, Sichuan Agricultural University (Ya’ an, China). Before the challenge test, the freezable bacterial solution was taken out at -80^°^C, thawed and mixed, and inoculated into the sterilized liquid medium at the ratio of 1/1,000, incubated at 37^°^C on a 180 r/min constant temperature oscillator for 10–12 h. The optical density (OD) value of the bacterial solution was measured at 600 nm, and the concentration of the bacterial solution was calculated according to the standard curve. Then the medium was used to adjust the concentration to 1.0 × 10^9^ CFU/ml. On day 7, the ducks in the APEC-challenged group were given a gavage of 0.5 ml of APEC suspension (1.0 × 10^9^ CFU/ml) and ducks in the unchallenged group were given a gavage of 0.5 ml of sterilized saline.

### Sample Collection

Sampling at 2 days post-infection (2 DPI) and 8 DPI, ducks were weighed after 8-h feed withdrawal, and the feed intake (FI) and mortality were recorded in detail on a per replicate basis. One experimental duck closest to the average weight of each replicate was selected and slaughtered by cervical dislocation. Blood was collected into no-additive blood collection vacuum tubes *via* the jugular vein and then centrifuged at 3500 *g* for 15 min at 4^°^C, taken serum into 1.5 ml microcentrifuge tube and stored at -20^°^C to determine biochemistry in serum. The thymus, spleen, and bursa of fabricius were removed intact and the surface blood stains were wiped off, then weighed and recorded. The middle segments (approximately 1–2 cm) of jejunum were excised for morphology analysis, removed the jejunal digesta, lightly rinsed with saline, and then the remaining jejunal tissue was divided into freezing tubes for testing the gene expression. In addition, at 8 DPI, cecal contents were aseptically collected into sterilized freezing tubes and assessed for enumeration of cecal bacteria.

### Measurements

#### Growth Performance

The experimental period was divided into two phases: 1–6 days (before APEC challenge) and 7–14 days (after APEC challenge). The BW, feed consumption, mortality, and feed loss of birds were recorded in detail by replicates. Then body weight gain (BWG), FI, and feed-to-weight ratio (F/G) were calculated for 1–6 days and 7–14 days, respectively. Mortality was only measured for 7-14 days.

#### Immune Organ Index

The thymus, spleen, and bursa of fabricius of each sampling duck were isolated and weighed. The relative organ weights were calculated by following the equation below:

Organ weight indexes = Immune organ weight (g)/Body weight (g) × 100

#### Immunoglobulins and Complement Components

The concentrations of serum immunoglobulin A (IgA), IgG, IgM, complement component 3 (C3), and C4 were measured by an enzymatic chromatometric method using the duck-specific ELISA Kits (Gersion Bio-Technology Co., Ltd., Beijing, China). The specific operation steps are strictly in accordance with the kit operation manual.

#### Intestinal Morphology Analysis

Jejunal tissue specimens were fixed in 4% (w/v) buffered paraformaldehyde for more than 48 h. Alcohol was used as dehydrating agent, and the tissue was dehydrated and placed in xylene for transparent treatment. Then the tissue was embedded in paraffin and cut into 5-μm thick slices and stained using hematoxylin and eosin (H&E). The slices were placed on slides for sectioning. Finally, the prepared tissue slices were used to measure villus height (VH) and crypt depth (CD) using a light microscope (BA400Digital, Mike Audi Industrial Group Co., Ltd., Xiamen, China) equipped with a digital camera with a magnification of 40×. Ten intact VH and the corresponding CD were measured using Image Pro Plus 6.0 (Rockville, MD, United States), and the mean value and V/C were calculated. The determination standard of VH and CD according to Nabuurs et al. (1993).

#### Quantitation of mRNA Using Real-Time Polymerase Chain Reaction

The total RNA of jejunum tissue was extracted by RNA-easy™ Isolation Reagent (Vazyme, Nanjing, China) according to the manufacturer’s instructions, and then the concentration was determined using NanoDrop 2000 (Thermo Fisher Scientific, Waltham, MA, United States), RNA purity was verified by the ratio of absorbance at 260 nm/280 nm. Next, the total RNA was reverse-transcribed into complementary DNA (cDNA) by HiScript ^®^ RT SuperMix for qPCR (+ gDNA wiper) (Vazyme, Nanjing, China) using the Bio-Rad DNA Engine (United States). The quantitative real-time polymerase chain reaction (RT-PCR) analysis was performed on CFX-96 Real-Time PCR System (Bio-Rad, United States) using ChamQ SYBR Color qPCR Master Mix (Vazyme, Nanjing, China). To construct a 10-μl reaction system, including 1 μl of cDNA template, 1 μl of reverse primer (4 μM), 1 μl of forward primer (4 μM), 2 μl of dd H_2_O and 5 μl of ChamQ SYBR Color qPCR Master Mix (2×). Primers of the target gene were designed with Primer 5.0 (Premier Biosoft International, Palo Alto, CA, United States) and listed in Table 2. All designed primers synthesized by GENEWIZ Biotechnology Co., Ltd. (Suzhou, China). β*-actin* were selected as the reference genes, and the relative mRNA abundance of target gene mRNA was calculated using the method of 2^–ΔΔ*Ct*^, as described by Livak and Schmittgen (2001).

**TABLE 2:** Primer sequences for quantitative RT-PCR.

Gene	Primer sequence (5′-3′)	Size (bp)	Accession no.
β*-actin*	Forward: AGAAATTGTGCGTGACATCAA Reverse: GGACTCCATACCCAAGAAAGAT	227	XM013108556.1
*IL-1*β	Forward: TCGACATCAACCAGAAGTGC Reverse: GAGCTTGTAGCCCTTGATGC	185	DQ393268
*IL-6*	Forward: CTGCGAGAACAGCATGGAGA Reverse: GAAAGGTGAAAAGCCCGCTG	191	XM_013100522.2
*IL-10*	Forward: GCTGGAGATGATGCGGTTCT Reverse: ATGTCAAACTCCCCCATCGC	179	XM_013092231.1
*IFN-*γ	Forward: TCGTGGAACTGTCAAACCTTCA Reverse: TTGGAAGTCGAAGTCTCCACC	101	XM_013106209.1
*TNF-*α	Forward: ACAGGACAGCCTATGCCAAC Reverse: ACAGGAAGGGCAACACATCT	165	XM_005019359.2
*TLR4*	Forward: ACCCATTGTCACCAACATCATC Reverse: TGCCTCAGCAAGGTCTTATTCA	195	JN048668.1
*Myd88*	Forward: GGAGGATGGTGGTCGTCATT Reverse: CCGCAGGATACTTGGGAACT	158	NM_001310832.1
*NF-*κ*B*	Forward: GCTGGCTAATTGGACCGACA Reverse: CAGGTCTGGCACGTATCTCG	122	XM_021271051.2
*ZO-1*	Forward: ACGCTGGTGAAATCAAGGAAGAA Reverse: AGGGACATTCAACAGCGTGGC	255	XM_013093747.1
*ZO-2*	Forward: ACAGTGAAAGAAGCTGGCGTAG Reverse: GCTGTATTCCCTGCTACGGTC	131	XM_005019888.2
*ZO-3*	Forward: CAACATCCCTGACATGGAAGACAT Reverse: TGTGTTCGTGTTGGTTGCGG	187	XM_013109403.1
*OCLN*	Forward: CAGGATGTGGCAGAGGAATACAA Reverse: CCTTGTCGTAGTCGCTCACCAT	160	XM 013109403.1
*CLDN2*	Forward: CTCCTCCTTGTTCACCCTCATC Reverse: GAACTCGCTCTTGGGTTTGTG	160	XM013104936.1
*sIgA*	Forward: TCGCTCAAGGAACCCATCGT Reverse: GCGGGACCACGAGAACTTCA	174	U27222.1
*AvBD2*	Forward: CCAGGTTTCTCCAGGATTGT Reverse: AACCCAAAGCAACTTCCAAC	117	AY641439.1
*AvBD16*	Forward: CGCTGCAGGAAACTCTGTC Reverse: CCCGAACATCTCCCAATATG	96	JQ359445.1

*IL-1β, Interleukin-1β; IL-6, Interleukin-6; IL-10, Interleukin-10; IFN-γ, Interferon-γ; TNF-α, Tumor necrosis factor-α; TLR4, Toll-like receptors 4; Myd88, Myeloid differentiation factor 88; NF-κB, Nuclear factor-kappa B; ZO-1, Zonula occludens-1; ZO-2, Zonula occludens-2; ZO-3, Zonula occludens-3; OCLN, Occludin; CLDN2, Claudin2; sIgA, Secretory immunoglobulin A; AvBD2, Avian β-defensin 2; AvBD16, Avian β-defensin 16.*

#### The Concentration of MUC2 in the Jejunum

The jejunal MUC2 concentration was measured by the duck-specific mucin 2 kit (Weskong Bioscience, Chengdu, China), the specific operation steps are strictly in accordance with the kit instructions.

#### Intestinal Microbial Population

The total microbial DNA in the cecal content was extracted by using the Stool DNA Kit (Omega Bio-Tech) according to the manufacturer’s instruction. The determination of purity and concentration of DNA by NanoDrop 2000. The populations of total bacteria, *Bacillus*, *Bifidobacterium*, *E. coli*, and *Lactobacillus* were determined by q-PCR. The primer and probe sequences used for q-PCR were obtained from previously literature (Wang et al., 2020) and synthesized by Thermo Fisher Scientific (Shanghai, China), and showed in Table 3. The PCR amplification of total bacteria by SYBR Green I chimeric fluorometry was performed in a 25 μl reaction mixture containing 1 μl of DNA template, 1.5 μl of each prime (10 μM), 9.5 μl dd H_2_O and 5 μl ChamQ SYBR Color qPCR Master Mix (2×). The q-PCR of *Bacillus*, *Bifidobacterium*, *E. coli* and *Lactobacillus* by TaqMan probe method, and to establish a 20 μl reaction system including 0.4 μl of fluorescent probe (10 μM), 0.6 μl of each prime (10 μM), 1 μl of DNA template, 7.4 μl dd H_2_O, 10 μl SuperReal PreMix (Probe, 2×) (Tiangen, Beijing, China). A specific standard curves were generated by constructing standard plasmids for bacterial quantification according to Chen et al. (2013). The data of bacterial quantification were analyzed after transformed (log_10_), and presented as total CFU/g digesta.

**TABLE 3:** Sequences of primers and probes for quantitative RT-PCR.

Items	Primer and probe sequences (5′–3′)	Size (bp)
Total bacteria	Forward: ACTCCTACGGGAGGCAGCAG Reverse: ATTACCGCGGCTGCTGG	200
*Bacillus*	Forward: GCAACGAGCGCAACCCTTGA Reverse: TCATCCCCACCTTCCTCCGGT Probe: CGGTTTGTCACCGGCAGTCACCT	92
*Bifidobacterium*	Forward: CGCGTCCGGTGTGAAAG Reverse: CTTCCCGATATCTACACATTCCA Probe: ATTCCACCGTTACACCGGGAA	121
*Escherichia coli*	Forward: ATTCCACCGTTACACCGGGAA Reverse: CGGGTAACGTCAATGAGCAAA Probe: AGGTATTAACTTTACTCCCTTCCTC	96
*Lactobacillus*	Forward: GAGGCAGCAGTAGGGAATCTTC Reverse: CAACAGTTACTCTGACACCCGTTCTTC Probe: AAGAAGGGTTTCGGCTCGTAAAACTCTGTT	126

#### Statistical Analysis

All data were analyzed as a completely randomized design with a factorial arrangement (4 × 2) using the GLM procedure of SPSS 25.0 (SPSS, Inc., Chicago, IL, Unites States). The model equation included main effects (APEC challenged and Zn–Met levels) and their interactions. When the main effects and their interactions were significant, differences among treatment means were tested using Duncan’s test. A *p*-value of less than 0.05 was used as the criterion of statistically significant. The results were expressed as means and pooled standard error of the mean.

## Results

### Growth Performance

As shown in Table 4 (1–6 days) and Table 5 (7–14 days). Dietary Zn–Met supplementation had no effect on the BW at 6 days of ducks, and BWG, FI and feed/gain (F/G) during 1–6 days of ducks (*p* > 0.05). However, APEC challenge significantly reduced BW at 14 days and BWG during 7–14 days, meanwhile markedly increased F/G and mortality during 7–14 days of ducks (*p* < 0.05). Ducks fed Zn–Met at 120-mg/kg diet had significantly higher BW at 14 days and BWG during 7–14 days, and lower mortality during 7–14 days of ducks (*p* < 0.05). There was an interaction between Zn–Met and APEC challenge observed in mortality. The C120 group had a dramatically lower mortality rate than C0 (*p* < 0.05).

**TABLE 4:** Effect of dietary Zn–Met levels on growth performance of duck during 1–6 days.

Zn level (mg/kg)	Body weight (g)		Body weight gain (g)	Feed intake (g)	Feed/Gain, F/G (g/g)
	1 day	6 days			
0	49.58	180.98	131.38	223.51	1.71
30	49.73	186.98	137.25	220.49	1.63
60	49.63	189.97	140.32	230.87	1.64
120	49.70	186.76	137.06	232.75	1.70
SEM	0.034	1.821	1.822	3.085	0.030
*P*-value	0.425	0.346	0.356	0.506	0.696

*In the same row, values with different letter superscripts mean significant difference (p < 0.05), and there a tendency was defined as 0.05 ≤ p < 0.10. The same as below.*

**TABLE 5:** Effect of dietary Zn–Met levels on growth performance of duck challenged with APEC from 7 to 14^1^ days.

Items		Body weight (g)	Body weight gain (g)	Feed intake (g)	Feed to gain ratio, F/G (g/g)	Mortality rate (%)
		14 days				
U0		615.27	434.67	688.44	1.61	1.67*^b^*
U30		639.56	450.96	712.86	1.59	3.33*^b^*
U60		657.64	465.26	725.61	1.56	0.00*^b^*
U120		686.30	499.91	767.67	1.54	3.33*^b^*
C0		556.53	376.34	632.79	1.69	31.67*^a^*
C30		593.43	410.22	672.00	1.63	25.00*^a^*
C60		606.62	414.00	673.01	1.66	26.67*^a^*
C120		638.20	451.08	732.49	1.62	8.70*^b^*
**Main effect**						
Zn level	0	585.90*^b^*	405.51*^b^*	660.62	1.65	16.67*^a^*
	30	616.49*^ab^*	430.59*^ab^*	692.43	1.61	14.17*^a^*
	60	632.13*^ab^*	439.63*^ab^*	699.31	1.61	13.33*^a^*
	120	662.25*^a^*	475.49*^a^*	750.08	1.58	6.02*^b^*
Challenge	-	649.69*^a^*	462.70*^a^*	723.65	1.58*^b^*	2.08*^b^*
	+	598.69*^b^*	412.91*^b^*	677.57	1.65*^a^*	23.01*^a^*
SEM		9.327	8.782	15.431	0.013	1.326
*p*-value						
Level		0.037	0.045	0.213	0.321	0.040
Challenge		0.010	0.008	0.144	0.008	<0.001
Level × Challenge		0.995	0.988	0.994	0.878	0.010

*^1^U0: control diet, U30: control + 30-mg/kg Zn–Met, U60: control + 60-mg/kg Zn–Met, U120: control + 120-mg/kg Zn–Met, C0: control + APEC, C30: control + APEC + 30 mg/kg Zn–Met, C60: control + APEC + 60-mg/kg Zn–Met, C120: control + APEC + 120-mg/kg Zn–Met. In the same row, values with different letter superscripts mean significant difference (P < 0.05).*

### Immune Organ Index

As demonstrated in Table 6, APEC challenge significantly decreased the thymus index at 2 DPI and bursa of fabricius index at 2 DPI and 8 DPI, but markedly increased the spleen index at 2 DPI of ducks (*p* < 0.05). Furthermore, dietary Zn–Met supplementation had an impact on thymus index at 2 DPI and 8 DPI. Ducks fed Zn–Met at 30, 60, and 120-mg/kg diets had a higher thymus index at 2 DPI compared to no Zn–Met supplementation ducks (*p* < 0.05), and the impact lasted in those fed diets with 120-mg/kg Zn–Met supplementation until the 8 DPI (*P* < 0.05). There were no significant interactions between Zn–Met and APEC challenge observed in immune organ index (textitp > 0.05).

**TABLE 6:** Effect of dietary Zn–Met levels on the relative weight of immune organs of meat duck challenged with APEC (%)^1^.

Items		2 DPI			8 DPI		
		TI	SI	BI	TI	SI	BI
U0		0.30	0.13	0.22	0.47	0.09	0.23
U30		0.51	0.12	0.20	0.61	0.10	0.21
U60		0.49	0.12	0.21	0.60	0.12	0.21
U120		0.46	0.11	0.20	0.73	0.11	0.20
C0		0.27	0.23	0.14	0.48	0.10	0.17
C30		0.34	0.22	0.16	0.53	0.12	0.18
C60		0.41	0.20	0.16	0.54	0.12	0.18
C120		0.43	0.16	0.19	0.59	0.12	0.18
**Main effect**							
Zn level	0	0.28^b^	0.18	0.18	0.47^b^	0.10	0.20
	30	0.43^a^	0.17	0.18	0.57^ab^	0.11	0.20
	60	0.45^a^	0.16	0.19	0.57^ab^	0.12	0.20
	120	0.45^a^	0.14	0.19	0.66^a^	0.11	0.19
Challenge	-	0.44^a^	0.12^b^	0.21^a^	0.60	0.11	0.21^a^
	+	0.36^b^	0.20^a^	0.16^b^	0.53	0.11	0.18^b^
SEM		0.017	0.008	0.007	0.019	0.005	0.008
*p*-value							
Level		0.004	0.295	0.819	0.014	0.451	0.994
Challenge		0.031	<0.001	0.004	0.085	0.510	0.038
Level × Challenge		0.494	0.730	0.512	0.514	0.930	0.902

*^1^TI, thymus index; SI, spleen index; BI, bursa of fabricius index. In the same row, values with different letter superscripts mean significant difference (P < 0.05).*

### Immunoglobulins and Complement Components

**FIGURE 1:**
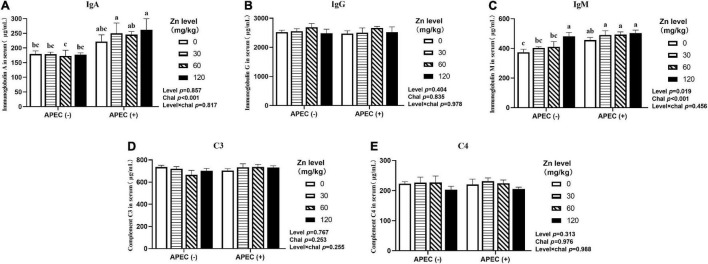
Effect of dietary Zn–Met levels on serum levels of IgA **(A)**, IgG **(B)**, IgM **(C)** and complement C3 **(D)** and C4 **(E)** of meat duck challenged with APEC O78 (2 DPI). Bars with different letters are statistically significant (*P* < 0.05). Results are expressed as mean ± SEM (*n* = 6).

**FIGURE 2:**
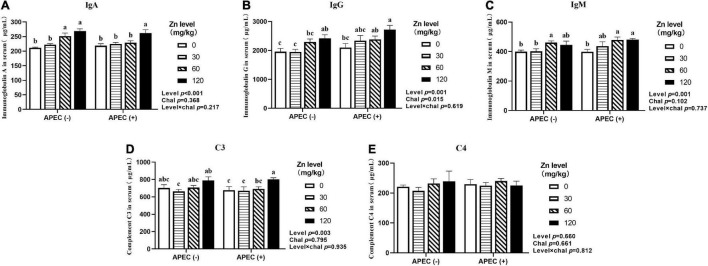
Effect of dietary Zn–Met levels on serum levels of IgA **(A)**, IgG **(B)**, IgM **(C)** and complement C3 **(D)** and C4 **(E)** meat duck challenged with APEC O78 (8 DPI). Bars with different letters are statistically significant (*P* < 0.05). Results are expressed as mean ± SEM (*n* = 6).

As illustrated in Figures 1, 2, APEC challenge sharply increased serum IgA, IgM levels at 2 DPI and IgG level at 8 DPI of ducks (*p* < 0.05). The serum IgA, IgG, IgM levels at 8 DPI were increased in ducks fed Zn–Met at 60 and 120-mg/kg diets compared to no Zn–Met supplementation ducks (*p* > 0.05). In addition, the serum IgM level at 2 DPI and C3 level at 8 DPI were increased at Zn–Met inclusion of 120-mg/kg (*p* > 0.05). There were no significant interactions between Zn–Met and APEC challenge observed in immunoglobulins and complement components (*p* > 0.05).

### Intestinal Morphology

**FIGURE 3:**
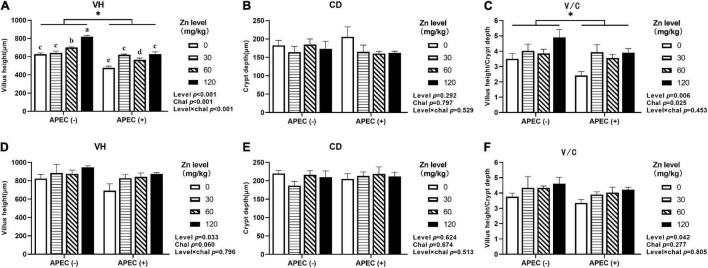
Effect of dietary Zn–Met levels on the jejunum VH **(A)**, CD **(B)**, and V/C **(C)** of meat duck challenged with APEC O78 (2 DPI). Effect of dietary Zn–Met levels on the jejunum VH **(D)**, CD **(E)**, and V/C **(F)** of meat duck challenged with APEC O78 (8 DPI). Bars with different letters are statistically significant (*P* < 0.05) and * means significant (*P* < 0.05) between APEC (−) and APEC (+). Results are expressed as mean ± SEM (*n* = 6).

**FIGURE 4:**
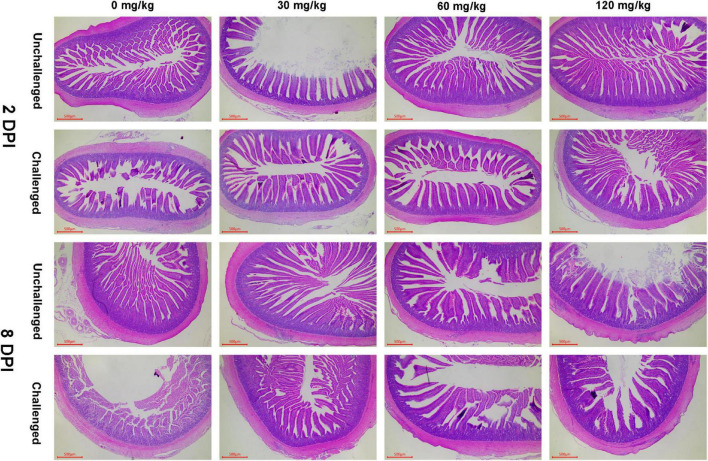
Effect of dietary Zn–Met levels on the jejunum morphology of meat duck challenged with APEC (HE stain). Results are expressed as mean ± SEM.

As shown in Figures 3, 4, APEC challenge dramatically reduced the jejunal VH and V/C at 2 DPI. The jejunal VH and V/C were gradually increased with the Zn–Met supplementation increased in both challenged and non-challenged groups (*p* < 0.05). The jejunal VH and V/C at 2 DPI and 8 DPI were improved in ducks fed Zn–Met at 120-mg/kg diet than that of those receiving no Zn–Met supplementation (*p* < 0.05). In addition, the jejunal VH was increased at Zn–Met inclusion of 30- and 60-mg/kg (*p* > 0.05). There was an interaction between Zn–Met and APEC challenge observed in the VH of jejunum at 2 DPI, which was higher in C30, C60, and C120 than C0 (*p* < 0.05).

### The mRNA Expression of Inflammatory Cytokines and *TLR4* Signaling Pathway in Jejunum

**FIGURE 5:**
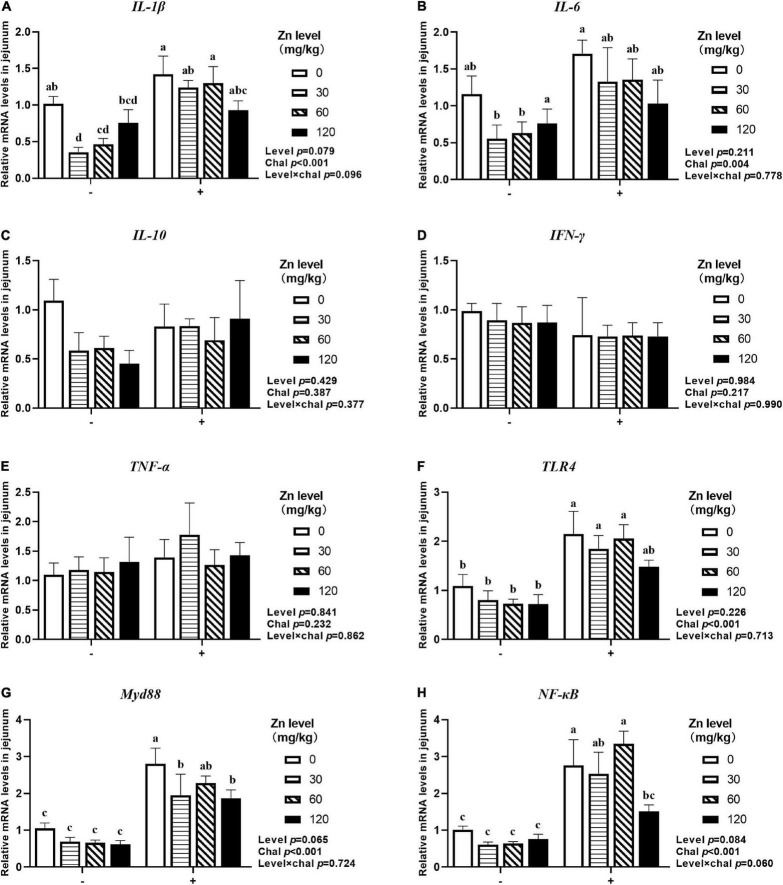
Effect of dietary Zn–Met levels on the mRNA expression of inflammatory cytokines *IL-1β* **(A)**, IL-6 **(B)**, *IL-10* **(C)**, *IFN-γ* **(D)**, *TNF-α* **(E)** and TLR4 signaling pathway related gene **(F–H)** in jejunum of meat duck challenged with APEC (2 DPI). Bars with different letters are statistically significant (*P* < 0.05). Results are expressed as mean ± SEM (*n* = 6).

**FIGURE 6:**
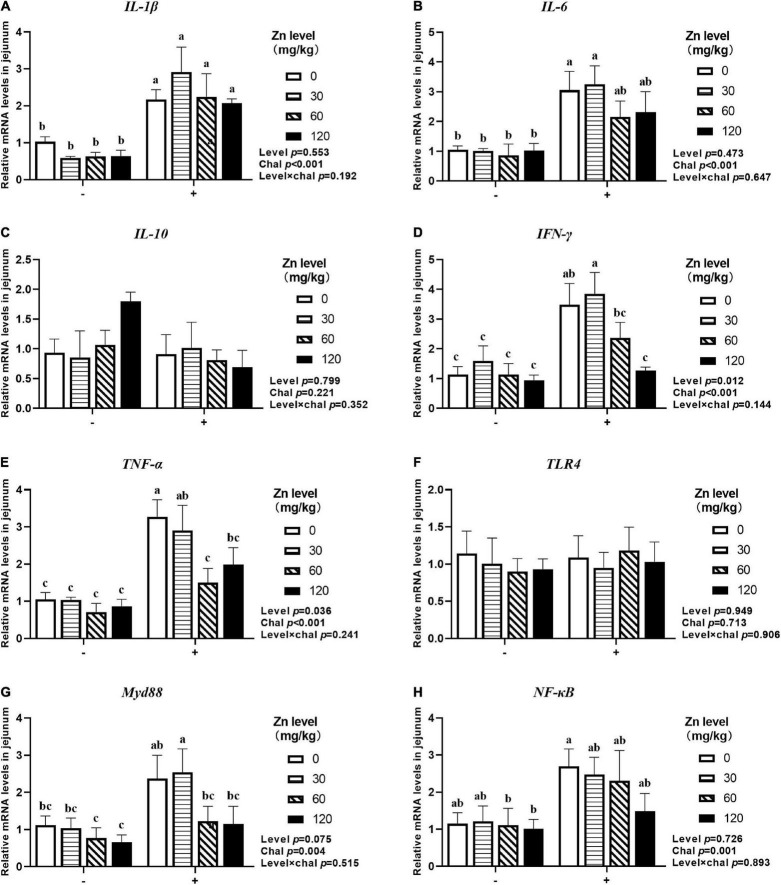
Effect of dietary Zn–Met levels on the mRNA expression of inflammatory cytokines *IL-1β* **(A)**, IL-6 **(B)**, *IL-10* **(C)**, *IFN-γ* **(D)**, *TNF-α* **(E)** and TLR4 signaling pathway related gene **(F–H)** in jejunum of meat duck challenged with APEC (8 DPI). Bars with different letters are statistically significant (*P* < 0.05). Results are expressed as mean ± SEM (*n* = 6).

As shown in Figures 5, 6, APEC challenge significantly upregulated *IL-1*β, *IL-6*, *TLR4*, *Myd88*, *NF-*κ*B* mRNA expression at 2 DPI and *IL-1*β, *IL-6*, *IFN-*γ, *TNF-*α, *Myd88*, *NF-*κ*B* mRNA expression at 8 DPI in jejunum (*p* < 0.05). At 8 DPI, the mRNA expression of *TNF-*α and *IFN-*γ were downregulated in the ducks fed Zn–Met at 60- and 120-mg/kg diets, respectively, than those fed diets without Zn–Met supplementation (*p* < 0.05). Furthermore, dietary Zn–Met supplementation tended to downregulate *IL-1*β (*p* = 0.079), *Myd88* (*p* = 0.065), *NF-*κ*B* (*p* = 0.084) at 2 DPI and *Myd88* (*p* = 0.075) mRNA expression at 8 DPI in jejunum. There were no significant interactions between Zn–Met and APEC challenge observed in the mRNA expression of inflammatory cytokines and *TLR4* signaling pathway in jejunum (*p* > 0.05).

### The mRNA Expression of Barrier-Related Gene in Jejunum

**FIGURE 7:**
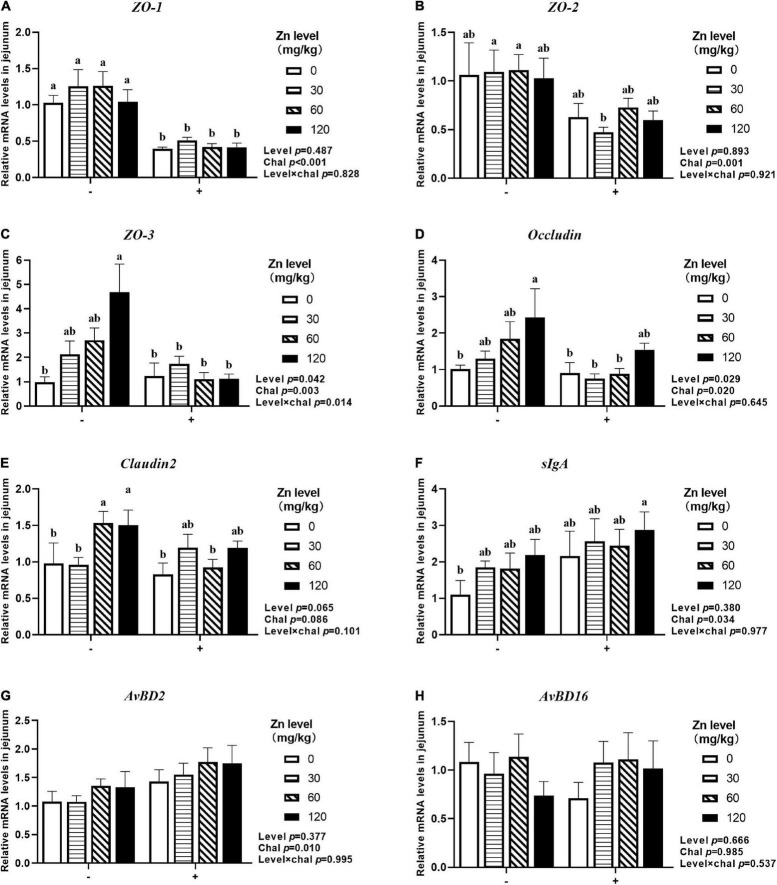
Effect of dietary Zn–Met levels on the mRNA expression of barrier-related gene *ZO-1* **(A)**, *ZO-2* **(B)**, *ZO-3* **(C)**, *Occludin* **(D)**, *Claudin2* **(E)**, *sIgA* **(F)**, *AvBD2* **(G)** and *AvBD16* **(H)** in jejunum of meat duck challenged with APEC (2 DPI). Bars with different letters are statistically significant (*P* < 0.05). Results are expressed as mean ± SEM (*n* = 6).

**FIGURE 8:**
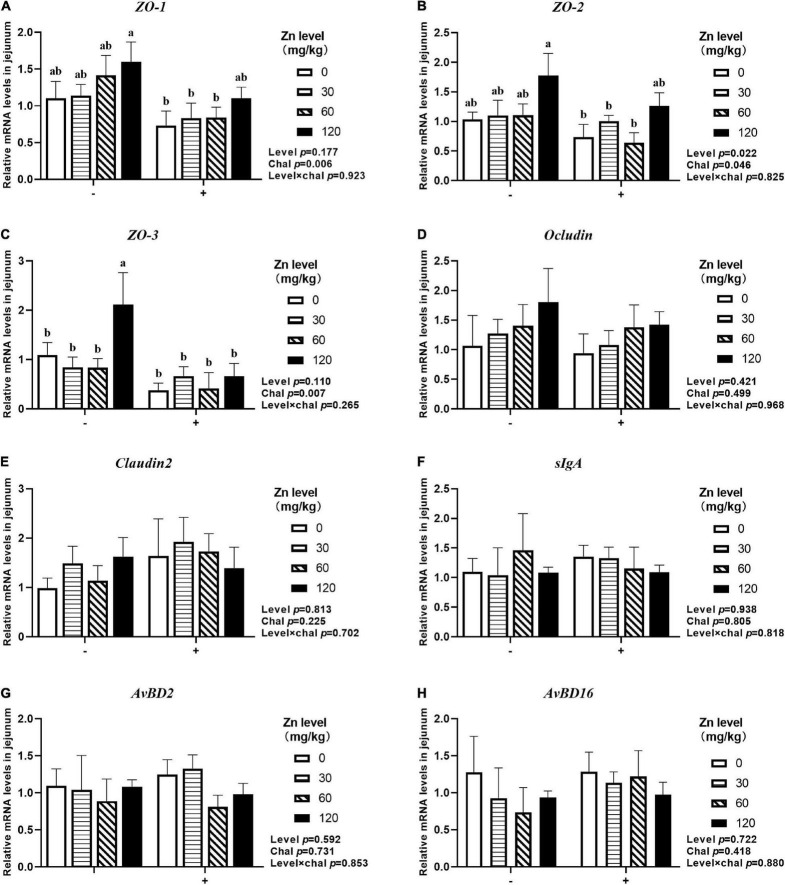
Effect of dietary Zn–Met levels on the mRNA expression of barrier-related gene *ZO-1* **(A)**, *ZO-2* **(B)**, *ZO-3* **(C)**, *Occludin* **(D)**, *Claudin2* **(E)**, *sIgA* **(F)**, *AvBD2* **(G)** and *AvBD16* **(H)** in jejunum of meat duck challenged with APEC (8 DPI). Bars with different letters are statistically significant (*P* < 0.05). Results are expressed as mean ± SEM (*n* = 6).

As shown in Figures 7, 8, APEC challenge significantly downregulated *ZO-1*, *ZO-2*, *ZO-3*, *OCLN* mRNA expression at 2 DPI and *ZO-1*, *ZO-2*, *ZO-3* mRNA expression at 8 DPI in jejunum (*p* < 0.05). At the same time, the jejunal mRNA expression of *sIgA* and *AvBD2* were upregulated after APEC challenge (*p* < 0.05). Ducks fed Zn–Met at 120-mg/kg diet had a higher *ZO-3, OCLN* mRNA expression at 2 DPI and *ZO-2, ZO-3* mRNA expression at 8 DPI compared to those fed diets without Zn–Met supplementation (*p* < 0.05). In addition, a dietary Zn–Met supplementation tended to upregulated *CLDN2* mRNA expression at 2 DPI (*p* = 0.065). The interactions between Zn–Met and APEC challenge had no influence on the mRNA expression of barrier-related gene in jejunum (*p* > 0.05).

### The MUC2 Concentration in Jejunum

**FIGURE 9:**
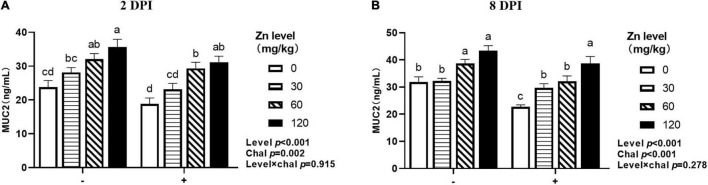
Effect of dietary Zn–Met levels on jejunum 2DPI MUC2 **(A)** and 8DPI MUC2 **(B)** concentration of meat duck challenged with APEC. Bars with different letters are statistically significant (*P* < 0.05). Results are expressed as mean ± SEM (*n* = 6).

As shown in Figure 9, both APEC challenge and Zn–Met had a significant effect on the MUC2 concentration in jejunum. Both at 2 DPI and 8 DPI, the jejunal MUC2 concentration were decreased after challenged with APEC (*p* < 0.05), and gradually increased with the Zn–Met supplementation increased (*p* < 0.05). At 2 DPI, the jejunal MUC2 concentration were increased in ducks fed Zn–Met at 30, 60, and 120-mg/kg diets compared to no Zn–Met supplementation ducks (*p* < 0.05). In addition, the jejunal MUC2 concentration at 8 DPI were increased at Zn–Met inclusion of 60- and 120-mg/kg diets (*p* > 0.05). There were no significant interactions between Zn–Met and APEC challenge observed in the MUC2 concentration in jejunum (*p* > 0.05).

### Intestinal Microbial Population

As shown in Table 7, both APEC challenge and Zn–Met had a significant impact on the populations of *Bifidobacterium*, *E. coli* and *Lactobacillus* in the cecal digesta. APEC challenge significantly increased the populations of *E. coli*, while decreasing the populations of *Bifidobacterium* and *Lactobacillus* (*p* < 0.05). On the contrary, an increase in *Bifidobacterium* and *Lactobacillus* populations, and a decrease in *E. coli* populations were found in ducks fed Zn–Met at 120-mg/kg diets when compared to no Zn–Met supplementation ducks (*p* < 0.05). There were no significant interaction effects on microbial population in the cecal digesta between dietary Zn–Met and APEC challenge (*p* > 0.05).

**TABLE 7:** Effect of dietary Zn–Met levels and APEC challenge on the cecal bacteria of meat ducks (8 DPI).

Items		Total bacteria	*Bacillus*	*Bifidobacterium*	*Escherichia coli*	*Lactobacillus*
U0		10.27	8.06	7.99	8.54	6.74
U30		10.31	8.18	8.18	7.37	7.50
U60		10.66	8.05	8.41	8.16	7.30
U120		10.46	7.95	9.04	7.57	7.88
C0		10.58	8.08	7.41	9.07	6.23
C30		10.49	8.03	7.78	8.50	6.79
C60		10.58	7.86	7.41	8.45	6.46
C120		10.60	7.73	8.08	8.25	7.37
**Main effect**						
Zn level	0	10.42	8.07	7.70^b^	8.80^a^	6.48*^c^*
	30	10.40	8.10	7.98^b^	7.94^b^	7.14^ab^
	60	10.62	7.95	7.91^b^	8.30^ab^	6.88^bc^
	120	10.53	7.84	8.56^a^	7.91^b^	7.62^a^
Challenge	-	10.42	8.06	8.40^a^	7.91^b^	7.36^a^
	+	10.56	7.92	7.67^b^	8.57^a^	6.71^b^
SEM		0.082	0.104	0.095	0.096	0.072
*p*-value						
Level		0.741	0.797	0.009	0.005	<0.001
Challenge		0.396	0.519	<0.001	0.001	<0.001
Level × Challenge		0.866	0.979	0.651	0.495	0.799

*In the same row, values with different letter superscripts mean significant difference (P < 0.05).*

## Discussion

To our knowledge, avian colibacillosis is triggered by APEC, induces a lot of deleterious effect, such as high mortality and decreased productivity, which imparts a severe economic loss on poultry production. The side effect of *E. coli* challenged on growth performance was reported in chicken (Zhang et al., 2014), piglet (Rodrigues et al., 2020), calf (Bi et al., 2017) and was confirmed in this study. In the present study, BW and BWG were reduced and mortality was sharply increased after APEC challenge, whereas dietary Zn–Met tended to alleviate the reduced BW and BWG. In addition, C120 significantly decreased mortality compared to C0. Although zinc is beneficial to animals in normal physiological states, there were only a few reports about Zn–Met on *E. coli* infection so far. Wei (2019) reported that dietary Zn–Met significantly improved growth performance by increasing ADG and decreasing the incidence of diarrhea of newborn dairy calves challenged with *E. coli*. Chen et al. (2019) also found weaned piglets fed with Zn–Met exhibited higher ADG and lower diarrhea rate after *E. coli* infection compares to control group. Furthermore, organic zinc exerts a protective role in other pathogens challenged model, such as coccidia (Bortoluzzi et al., 2019) and *Salmonella* (2014). The promotion effect on growth performance may be attributed to the fact that Zn–Met, as a source of organic zinc can be absorbed more efficiently than inorganic zinc, especially in case of impaired intestine (Yu et al., 2017; Wu et al., 2019). However, further investigations are needed to validate the results of this study due to the lack of literature about the effect of Zn–Met on *E. coli* challenged model in poultry.

The immune organ index is one of the important indicators to evaluate the development status of immune organs and immune function of poultry, birds with heavier immune organ usually show stronger immunity (Jin et al., 2019). The thymus is the site of T-cell differentiation and maturation, and plays an important role in the establishment of the immune function (Miller, 2002). The spleen, as an important peripheral lymphoid organ, has a filtering effect on all types of tumor cells and harmful microorganisms (Lewis et al., 2019). The bursa of fabricius is a unique immune organ of birds and plays a vital role in the development of the specific B-lymphocyte lineage which produced antibody in birds (Nera et al., 2015). Consistent with the previous studies (Zhang et al., 2020), a reduction of both thymus and bursa of fabricius index induced by APEC challenge in the current study indicated an obstruction of development of immune organs, whereas the increase in spleen index may be due to congestion and enlargement of spleen result from colibacillosis (Wani et al., 2017). Moreover, in our study, Zn–Met had a promotional effect on thymus organogenesis to combat the adverse impact on thymus ensued from APEC infection. A similar study showed that the weight of the spleen and bursa of fabricius is higher in *Eimeria tenella* infected broilers supplemented with ZnSO_4_ (Sajadifar, 2013). It may be explained by Zn is a pro-mitogen that can promote cell mitosis and affect the differentiation and growth of lymphocytes, while Zn deficiency restrains the development of lymphoid organs and the maturity and population of peripheral blood T-lymphocytes (Cui et al., 2004). These results suggest Zn–Met has a positive effect on immune organ development under challenge.

Owing to the important role in the immune system, immunoglobulins, and complement components are commonly used to evaluate the immune status of animals, and three main types, IgA, IgG, and IgM, are present in birds (Zhang et al., 2014). Immunoglobulin A plays a key role in defending mucosal surfaces against attack by infectious microorganisms (de Sousa-Pereira and Woof, 2019). Immunoglobulin G is the only immunoglobulin that can be transmitted to the offspring and function in recognition of antigens on the surfaces of invading viruses and bacteria and recruitment of effector molecules (Yanaka et al., 2020). Immunoglobulin M, as the first antibody in response to an invading pathogen, plays an important role in combating microbial infection and regulating the immune response (Gong and Ruprecht, 2020). The complement system consists of a tightly regulated network of proteins that play a key role in host homeostasis, inflammation, and in the defense against pathogens (Sarma and Ward, 2011). In this study, APEC challenge significantly increased serum IgA, IgM, and IgG, which suggests that the immune system responds to pathogen invasion even when immune organ is impaired. Similar finding was reported in previous studies by Zhang et al. (2014) in broilers. Zinc is necessary for maintenance of phagocytosis of macrophages and neutrophils (Driessen et al., 1994), and Kidd et al. (1994) found Zn–Met boosted clearance of *E. coli* from blood as a result of enhanced leading to a reduction of immune load. We also found that supplemental Zn–Met resulted in increased IgA, IgM, IgG, and C3 in serum. Our results are consistent with a report by Hamidi and Pourreza (2009) who observed increased serum IgA and antibody response to sheep red blood cell (SRBC) in mixed coccidial infected broilers fed with 60-mg/kg Zn–Met. In another study, Akhavan-Salamat and Ghasemi (2019) also found improvement of total and IgG antibody titers against SRBC for primary responses in Zn–Met supplemented broilers under high ambient temperature condition compared to birds fed ZnO, but no differences were observed in secondary responses. These results suggest that Zn–Met could exert a protective role in controlling APEC infection by improving immunity.

Villi height, CD and villi height/CD are the most widely used as a marker to evaluate the digestive and absorptive capacity of the gut and the maturation rate of intestinal epithelial cells (Lamb-Rosteski et al., 2008). Increasing the villi height increases and decreasing the CD together improve the nutrient absorption capability (Chwen et al., 2013). Villous atrophy and intestinal morphology disruption in broiler chickens challenged with *E. coli* (Mohebodini et al., 2019). Our study showed that APEC challenge decreased the jejunal VH and V/C, indicated a disturbance of intestinal villi-crypt structure. Furthermore, dietary Zn–Met supplementation increased the jejunal VH and V/C at 2 DPI and 8 DPI whether with or without APEC challenge. Similar studies have shown that the VH and V/C enhanced when feeding 120-mg/kg Zn in broilers challenged with *Salmonella* (Shao et al., 2014). Previous studies *in vitro* found that Zinc may promote intestinal epithelial wound healing by enhancement of epithelial cell restitution through a TGFβ-independent mechanism (Cario et al., 2000). In addition, zinc can effectively inhibit apoptosis by suppressing oxidative stress and death receptor-mediated pathways (Zhou et al., 2008). These results indicate that zinc can repair the intestinal injury resulted from APEC infection in meat ducks.

Inflammation is a pervasive phenomenon coordinated by multiple cytokines and immune signaling molecules that can arise in response to infection, injury and other adverse factors (Ashley et al., 2012). Although inflammation is a normal process for repair of tissue after an injury and cytokines are the bridge of cell–cell communications, uncontrolled inflammation represented by cytokine storm causes unwarranted energy wastage, irreversible organ damage, and unbearable pain (Gupta et al., 2019). Infections caused by pathogens are usually accompanied by a surge in pro-inflammatory cytokines, such as *IL-1*β and *IL-6* (Oh et al., 2019). In this study, the mRNA expression of *IL-1*β, *IL-6*, *IFN-*γ, and *TNF-*α was significantly upregulated by APEC challenge compared with unchallenged group, which is consistent with the observations in broiler chickens (Ateya et al., 2019) and piglets (Wan et al., 2019). Meanwhile, dietary Zn–Met supplementation tended to downregulate *IL-1*β (*p* = 0.061) at 2 DPI. Similarly, Bortoluzzi et al. (2019) reported that organic zinc supplementation downregulated *IL-8* mRNA expression and upregulated *IL-8* in jejunum of broiler chickens challenged with coccidia plus *Clostridium perfringens*. Moreover, organic zinc was found to be more effective in reducing serum *IL-1*β concentration of hens after administered with lipopolysaccharide (LPS) compared to ZnSO_4_ (Cheng and Guo, 2004).

Pattern recognition receptors (PRRs), which are specific molecules on host cells that recognize PAMPs, the specific target molecules of pathogens, and TLRs are crucial members of the PRRs (Kawai and Akira, 2010). Generally, *TLR4* primarily recognizes LPS in the cell wall of host extracellular pathogens, and *E. coli* can produce a variety of toxins, including LPS. When *TLR4* is activated, the cytosolic TIR domain of *TLR4* binds to myeloid differentiation factor 88 (MyD88) and then activates *NF-*κ*B* through cascade reaction, which eventually induces the expression of pro-inflammatory cytokines (such as *IL-1*β, *IL-6*, *TNF*α), chemokines, and immune co-stimulatory molecules (Liu et al., 2017). We found that, the mRNA expression of *TLR4*, *Myd88* and *NF-*κ*B* increased in APEC challenge ducks compared to non-infected ducks, which is similar to Huang et al. (2019) who reported that the mRNA and protein abundance of *TLR4*, *Myd88*, *NF-*κ*B* in jejunum and ileum in *E. coli*-challenged piglets. Zn–Met supplementation tended to downregulated the mRNA expression of *TLR4* and *NF-*κ*B* in jejunum. The results are similar to previous studies (Hu et al., 2017), it was found that CS-Zn supplementation downregulated the protein expression of *TLR4* and its downstream signals *NF-*κ*B*, *IKK*β, and *I*κ*B*α, along with a reduction of *IL-2*, *TNF-*α, *and IFN-*γ. According to previous studies, zinc acts as a crucial immune mediator against infection and inflammation, zinc deficiency enhances the generation of *IL-1*β and *TNF-*α in myeloid cells *via* epigenetic effects (Wessels et al., 2013). Zinc supplementation inhibited the activation of *NF-*κ*B* by upregulating the gene expression of A20 and the binding of its transactivating factor to DNA, leading to decreased production of inflammatory cytokines (Prasad et al., 2011). Combined with a reduced expression of pro-inflammatory cytokines, it can be concluded that Zn–Met alleviates the inflammatory response by reducing the expression of inflammatory cytokines possibly through suppressing the *TLR4*/*MyD88*/*NF-*κ*B* pathway, which may partially explain the reduction of mortality.

The intestinal barrier ensures the entrance of essential nutrients from the intestinal lumen and also effectively prevents harmful substances in the intestinal lumen (such as bacteria and various toxins) from entering the blood circulation and internal organs through the intestinal mucosa (Vancamelbeke and Vermeire, 2017). The barrier is consisted of physical, chemical, immunological, and microbiological components. The impaired intestinal barrier allows pathogens and toxins to enter the blood circulation more easily, thus causing the translocation of bacteria and toxins, which can easily lead to enterogenic infections and even to systemic inflammatory responses or multi-organ failure (Wageha et al., 2017). The physical barrier is composed of intestinal epithelium and junctional complexes, which mainly include desmosomes, adherens junctions (also known as zonula adherens), and tight junctions (such as occludin and claudins) (Groschwitz and Hogan, 2009). In this study, the mRNA expression of *ZO-1*, *ZO-2*, *ZO-3*, *OCLN* were downregulated in jejunum after APEC challenge, indicating the destruction of intestinal physical barrier, which is consistent with report of Gao et al. (2013) and Yang et al. (2014). Additionally, we found that dietary supplementation of Zn–Met to meat ducks challenged by APEC remarkably reversed the physical barrier injury to a certain extent by the improved level of *ZO-2*, *ZO-3* and *OCLN*. This was consistent with the report of Zhang et al. (2012) that Zn improved relative expression of *Claudin-1* and *OCLN* in ileum of broiler chickens challenged with *Salmonella*.

Secretory IgA (*sIgA*) secreted by epithelial cell is the main effector of the immunological barrier, its role is not only in the protection of the intestinal epithelium from enteric pathogens and toxins, but is also important for communication with gut-associated lymphoid tissue and restoration of homeostasis in pathology (Corthésy, 2013). Avian β-defensins (AvBD) are important components of host defense peptides (HDPs) that targeting a broad spectrum of pathogens through direct binding and lysis of microbial membranes, and neutralizing bacterial endotoxins (Dijk et al., 2008). Several HDPs possess different degrees of antibacterial activity for *E. coli*, *S. Enteritidis*, *S. Typhimurium*, *C. jejuni*, *C. perfringens in vitro* and protection from early chick mortality caused by APEC (Nguyen et al., 2021). The APEC challenge stimulated the mRNA expression of *sIgA* and *AvBD2*, which are similar to Cao et al. (2013) and Li et al. (2016). Zn–Met supplementation had no effects on the mRNA expression of *sIgA* and *AvBD2*, but Bun et al. (2011) found that organic zinc addition increased the cecal concentration of sIgA in broilers challenged with *E. tenella* compared to the control. The discrepancy may result from the difference of zinc sources and pathogens.

Mucin 2 (MUC2) is the major gel-forming mucin of the chemical barrier, which serves as a biophysical layer against the luminal hostile environment (Liu et al., 2020). The jejunal MUC2 concentration reduced after APEC challenged, the result is consistent with the report of Lee et al. (2017) that a reduction of ileal MUC2 mRNA expression in LPS-induced broiler chicken. Meanwhile, the jejunal MUC2 concentration increased with the level of Zn–Met supplementation in ducks whether APEC challenge or not. Similar study was reported by Wen et al. (2018) who found that ZnSO_4_ supplementation markedly upregulated the MUC2 mRNA expression of jejunum in Pekin ducks. But Bortoluzzi et al. (2019) showed that neither ZnSO_4_ nor organic Zn had an effect on the MUC2 mRNA expression in the jejunum of broiler chickens challenged with coccidia plus *C. perfringens*. The reduction of MUC2 secretion may be due to a decrease in the number goblet cells at early infection, however, MUC secretion is increasing with the restoration of immune function and the positive effect of zinc (Kim et al., 2015; Zhang et al., 2016). A study *in vitro* found that Zn supplementation enhanced intestinal barrier function by activating of PI3K/AKT/mTOR signaling. In addition, Song et al. (2015) reported that ZnO improved intestinal barrier function of weaned pigs by activating the ERK1/2 signaling pathway and inhibiting the JNK and p38 signaling pathway. So, more investigations are needed to elucidate the underlying mechanism of the effects of zinc on intestinal barrier.

Numerous commensal microbiota inhabited in the gastrointestinal tract can not only participate in the digestion and absorption of nutrients, but also plays a crucial role in the prevention and resolution of pathogenic infections. On the one hand, microbiota can resist pathogens by producing bacteriocins or microcins with antibacterial and bactericidal activity in a direct manner. On the other hand, microbiota defense against intestinal pathogens by regulating the processes of differentiation of T-cells, B-cell development, and antibody production in an indirect manner (Ubeda et al., 2017). Intestinal microbiota homeostasis is disrupted after pathogen infection, which may affect animal growth (Chen et al., 2020). In the current study, the cecal microbial population were measured by q-PCR. It was observed that the number of *E. coli* was increased, while the number of *Bifidobacterium* and *Lactobacillus* was reduced after APEC infection, which is similar to Zhang et al. (2014) reported in broiler chickens, indicating the out-of-balance of intestinal microbiota in meat ducks. Dietary Zn plays an important role in modulating the gut microbiota, massive studies have shown that pharmacological dose of Zn possess a positive effect on concentrations of short chain fatty acids, lactic acid bacterial, and other anaerobic bacterial growth, bacterial richness and diversity (Xia et al., 2017; Sun et al., 2019). The Zn deficiency leads to distinct taxonomic changes and reduction of overall species richness and diversity, establishing a microbial profile resembling that of various other pathological states (Spenser et al., 2015). In this study, we found that Zn–Met supplementation reversed the deleterious effect of APEC challenge by increasing *Bifidobacterium* and *Lactobacillus* counts, and decreasing *E. coli* counts. Shao et al. (2014) demonstrated that Zn supplementation increased the populations of the total bacteria and *Lactobacillus* group in cecal contents of *Salmonella*-infected broilers. Through 16S rRNA sequencing analyses, Feng et al. (2020) reported that dietary chitosan-chelated zinc (CS-Zn) reduced the relative abundance of *Proteobacteria*, as a potential diagnostic signature of dysbiosis and risk of disease, at the phylum level, while decreased the relative abundance of *Desulfovibrio*, *Peptococcus*, and increased *Lactobacillus*, *Romboutsia*, *Anaerotruncus* at the genera level in rats challenged with *E. coli*. Further research found the function of enrichment of carbohydrate metabolism and the metabolism of cofactors and vitamins were dramatically increased in CS-Zn treatment with the change of intestinal flora. However, our results did not evaluate gut microbiota community structure from different taxonomic levels, which should be further investigated.

## Conclusion

In conclusion, dietary Zn–Met supplementation alleviated the deleterious effects induced by APEC challenge. Inclusion of 120-mg/kg Zn–Met enhanced immune function by stimulating the development of immune organ, secretion of immunoglobulins, and promoted intestinal health by improving intestinal barrier downregulating of pro-inflammatory cytokines and regulating of the cecal microbiota, ultimately improving the growth performance in APEC-challenged meat ducks.

## Data Availability Statement

The original contributions presented in the study are included in the article/supplementary material, further inquiries can be directed to the corresponding author/s.

## Ethics Statement

The animal study was reviewed and approved by the Animal Health and Care Committee of Sichuan Agricultural University (No. 20180718).

## Author Contributions

JM: conceptualization, methodology, and writing original draft preparation. YC and ZZ: software, data curation, and revising the manuscript. TY, FW, and HZ: visualization and investigation. BW, GL, XC, GT, and JC: supervision, software, and validation. GJ: writing–reviewing and editing. All authors contributed to the article and approved the submitted version.

## Conflict of Interest

BW was employed by the company Chelota Group. The remaining authors declare that the research was conducted in the absence of any commercial or financial relationships that could be construed as a potential conflict of interest.

## Publisher’s Note

All claims expressed in this article are solely those of the authors and do not necessarily represent those of their affiliated organizations, or those of the publisher, the editors and the reviewers. Any product that may be evaluated in this article, or claim that may be made by its manufacturer, is not guaranteed or endorsed by the publisher.
